# Nutritional perspectives in female soccer: a scoping review

**DOI:** 10.1080/15502783.2024.2366427

**Published:** 2024-07-03

**Authors:** Diogo V. Martinho, Adam Field, Robert Naughton, Alex S. Ribeiro, André Rebelo, Élvio R. Gouveia, Hugo Sarmento

**Affiliations:** a University of Coimbra, Research Unit for Sport and Physical Activity, Faculty of Sport Sciences and Physical Education, Coimbra, Portugal; b Interactive Technologies Institute, Laboratory of Robotics and Engineering Systems, Funchal, Portugal; c Manchester Metropolitan University, Department of Sport and Exercise Science, Institute of Sport, Manchester, UK; d University of Huddersfield, School of Human and Health Sciences,Huddersfield, UK; e Universidade Lusófona, CIDEFES, Centro de Investigação em Desporto, Educação Física e Exercício e Saúde, Lisboa, Portugal; f COD, Center of Sports Optimization, Sporting Clube de Portugal, Lisboa, Portugal; g University of Madeira, Department of Physical Education and Sport, Funchal, Portugal

**Keywords:** Energy balance, low energy availability, carbohydrates, women soccer

## Abstract

**Background:**

The purpose of the review was to evaluate the literature exploring nutritional habits and practices in female soccer players.

**Methods:**

The PRISMA-ScR Items for Systematic Reviews and Meta-Analyses extension for Scoping Reviews were followed. Searches of Web of Science, PubMed and Scopus databases were conducted for studies exploring the nutritional habits and practices of female soccer players.

**Results:**

A total of 72 studies were included in the scoping review. Studies on female soccer players mainly focused on daily energy expenditure, daily energy and macronutrient intake and hydration status. A negative energy balance was consistent across studies, and the ingestion of CHO appears below the current recommendations. Female soccer players are predominately in negative energy balance, which may indicate that they are at risk of low energy availability. A high use of nutritional supplements is apparent in female soccer, whilst a large proportion of players commence training dehydrated.

**Conclusions:**

The current findings have implications for practitioners relating to the planning, management, monitoring, and implementation of nutritional intake and training and competition schedules.

## 1. Background

The popularity and professionalism of female soccer has increased markedly in recent years across all levels of the game. Data from the Fédération Internationale de Football Association (FIFA) obtained between 2015 to 2019 also reported an increase in female soccer players in different countries. It is estimated that 13 million females are playing organized soccer, and 63,000 coaches are working with female teams [1]. The rise in popularity has been accompanied with a 6-fold increase in published research papers [2]. The majority of research in female soccer involves observational studies objectively quantifying the match demands in female soccer [3–5]. However, there are fewer studies exploring the nutritional habits of female soccer players. Therefore, it is likely that most of what is currently known about and adopted in relation to female soccer is derived from observations or practices that have demonstrated efficacy in male soccer athletes. This could have detrimental implications for female soccer given that females possess different physiological profiles [6,7] that are likely to influence dietary needs between sexes.

International female soccer players competing in European leagues complete approximately 10 km total distance, 2.5 km high-speed distance (> 14.4 km.h^−1^) [3], 174 accelerations and 20 sprints (168 m) during official matches [8]. Training and competition demands are on an upwards rise in elite female soccer players. Consequently, this will lead to greater energy demands and the need for dietary modification to meet such demands. However, since there is a lack of empirical projects exploring the current landscape in female soccer, energy demands and nutritional habits are not fully understood. Players that fail to meet daily energetic and nutritional recommendations are at risk of low energy availability, commonly defined as < 30 kcal^−1.^kg_FFM_^−1.^day. Such low energy intake is related to adverse health effects, such as compromised bone health, irregular menstruation, and/or reduced immune functioning [9]. Therefore, it is key that a balanced appraisal of the literature is carried out in relation to the nutritional habits of female soccer players.

Energy availability and energy balance are similar but distinct terms, with the limitations of these concepts widely debated in the literature [10]. Energy availability represents the energy to sustain physiological functions after the cost of exercise has been met, and is calculated as energy intake minus exercise energy expenditure, expressed relative to fat-free mass [11,12]. Energy balance occurs when energy intake equals energy expenditure and is affected by numerous components of energy expenditure, including, exercise energy expenditure, resting metabolic rate, and dietary – and cold-induced thermogenesis, with this concept calculated via the subtraction of energy expenditure from energy input [13]. Previous studies have demonstrated that daily energy expenditure remains higher than daily energy intake in elite English [14] and Dutch players [15], indicative of a negative energy balance. Evidence suggests that soccer players tend not to meet the general recommendations for CHO intake in male soccer [16]. Given the negative implications of being in negative energy balance, particularly in females [12], characterizing the nutritional practices in this population remains a key priority.

Given the increasing popularity of female soccer, it is perhaps surprising that reviews summarizing the nutritional practices are scarce. A recent narrative review analyzed daily energy and macronutrient intake of seven studies using elite female soccer athletes [17]. However, there are no scoping reviews that have undertaken a preliminary assessment of the potential size and scope of the available research relating to energy and nutritional behaviors, dietary habits and supplementation in female soccer players. Furthermore, the heterogeneity of player age (16 − 41 years), stature (1.48 − 1.87 m) and mass (46 − 88 kg) among top-level players indicates that studies of a range of competitive groups and youth athletes should also be considered [18]. Therefore, this scoping review investigated nutritional habits and practices, including nutritional periodization, micronutrient intake and supplementation use, among female soccer players.

## 2. Methods

The present scoping review followed the Preferred Reporting Items for Systematic reviews and Meta-Analyses extension for Scoping Reviews (PRISMA-ScR) Checklist [19]. The protocol was developed by the research team and registered on the Open Science Framework at d oi:10.17605/OSF.IO/2YU3D.

### 2.1 Eligibility criteria

The inclusion criteria of the current scoping review were defined based on the type of participants, concept, context and types of evidence sources [20]. Studies that involved female youth or adult populations participating in competitive soccer were considered for the present review. The concept explored studies that investigated nutritional issues, dietary habits and supplementation of female soccer players. Types of evidence contained original research or dissertations written in English, Portuguese or Spanish. Reviews, case studies and letters to the editor were not analyzed. There were no defined restrictions regarding the year of publication or geographical location.

### 2.2 Information sources and search

The search strategy was divided into three different phases [21]. A preliminary search relating to nutrition in female soccer was conducted using PubMed. The titles and abstracts were originally screened and selected by two independent authors (DVM/HS). Three databases were consulted (i.e. Web of Science all databases, PubMed and Scopus) on 20 November 2022 and updated on 2 April 2024. The search results were exported into a reference manager software (EndNote X9; Thomson Reuters©, New York, NY, USA). Duplicates were automatically removed and manually checked to ensure that duplicates were removed. Manual searches of each study of the bibliography extracted from the first search were carried out to identify additional studies. Searches included the following keywords: *football* OR *soccer* AND *female* OR *women* AND nutrition* OR *nutritional status* OR *nutritional intake* OR *intake* OR *ingestion* OR nutrition habit* OR diet* OR *energy expenditure* OR *energy intake* OR *dietary intake* OR *dietary status* OR *dietary supplement* OR ergogenic aid* OR macronutrient* OR micronutrient*.

### 2.3 Selection of sources of evidence

The references were exported to EndNote which were assessed by two authors (DVM/AR). Records that did not follow the eligibility criteria in terms of population, context, content and types of evidence were initially excluded according to the titles and abstracts. Full-texts were consulted to identify studies that met the inclusion criteria. The screening of references and citations was also performed by two independent observers (DVM/AR) and disagreements between authors were solved by a third independent researcher (HS). Manuscripts were omitted if they violated any of the following criteria: involved male soccer players, included school or American, Australian or Gaelic football, contained no relevant data about nutritional aspects.

### 2.4 Data charting process, data items and synthesis

A data-charting sheet created on Microsoft Excel summarized the following parameters: authors, year of publication, journal, country, aim, sample characteristics (age, stature, mass, body composition, main results, limitations, conclusions and practical applications). Some of the information extracted from the manuscripts was not presented in the results section, but was retrieved to gain a comprehensive depiction of the study. Two authors (DVM/AR) independently extracted the relevant details, and the final information of studies included in the present review was confirmed by consensus. Study characteristics were summarized in tables according to the following topics: daily energy (intake and expenditure), fluid balance, supplementation, nutritional knowledge, other topics.

## 3. Results

### 3.1 Sources of evidence

**Figure 1.**
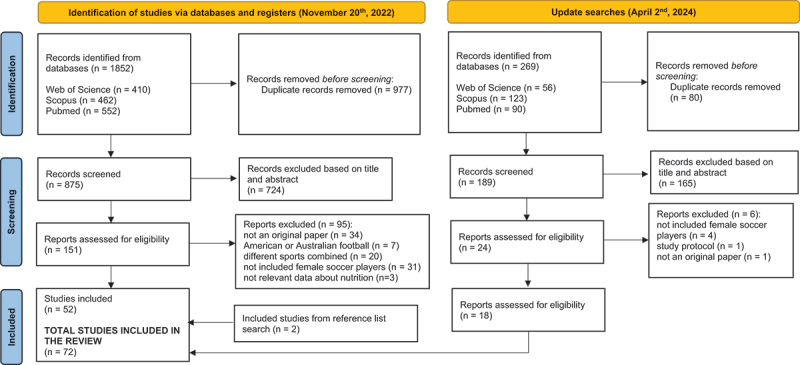
Flowchart of the review process.

During the initial searches, 52 studies were identified as eligible for the current review, with two additional articles located by the authors during manual searches. The updated searches identified 18 manuscripts that met the eligibility criteria. As a result, 72 papers were included in the scoping review (Figure 1).

### 3.2 Characteristics of sources of evidence

As shown in Table 1, female soccer players from the United States were included in 17 studies (24%), eight studies involved Polish players (11%), and seven studies contained soccer participants from the United Kingdom (~10%). Studies were mainly developed using adult participants (69%), while 18% were conducted with female youth participants (mean age < 18 years). Excluding the youth populations, 40% of studies involved participants competing at national level, 25% were university players and 13 studies incorporated elite players (22%).

**Table 1.** Characteristics and main conclusions of the studies included in the current review.

Study	Main topic	Country	Participants information	Methods	Key findings
Fogellhom et al. [22]	Energy expenditure/ dietary intake	Finland	N = 12 National league age (years): 18.5 ± 2.3 weight (kg): 60.8 ± 5.9 body fat (%): 25.8 ± 3.0 height (cm): 167.0 ± 5.0	Body composition assessed by underwater weighing and DXA, REE measured by indirect calorimetry, daily EE and EI estimated using 7-day records.	EE of female soccer players was, on average, 2251 kcal.day^−1^ while, EI was 2143 kcal.day^−1^. Consequently, an energy deficit of – 112 kcal.day^−1^ was noted. REE was, on average, 1383 kcal kcal.day^−1^.
Broad et al. [23]	Fluid intake	Australia	N = 17 Elite age (years): 16–28 weight (kg): 62.8 ± 8.5 skinfolds (mm): 93.6 ± 32.0 height (cm): 166.9 ± 7.1	Changes in body weight, fluid intake, urine and fecal outputs	No significant changes were observed in sweat rates between training and competitive period. Players were below the recommendations of fluid intake for women soccer (600 ml.hour^−1 –^ 1000 ml.hour^−1^)
Larson-Meyer et al. [24]	Supplementation – creatine	UK	N = 14 University placebo (N = 7) age (years): 19.0 ± 1.5 weight (kg): 66.3 ± 3.4 body fat (%): 25.8 ± 3.5 creatine (N = 7) age (years): 19.3 ± 1.47 weight (kg): 61.9 ± 5.3 body fat (%): 25.1 ± 6.5	Double-blind randomized placebo trial. Baseline measurements were obtained 1-2 weeks before the beginning of supplementation and, at 5 and 13 weeks (maximal strength and vertical jump). Body composition was analyzed using DXA. Off-season training consists in strength and soccer sessions.	The results of the current study showed creatine ingestion had potential beneficial effects on maximal strength but, the effects were not evident for body composition.
Cox et al. [25]	Supplementation – creatine	Australia	N = 12 Elite age (years): 22.0 ± 5.4 weight (kg): 62.6 ± 6.4 skinfolds (mm): 97.3 ± 27.9 height (cm): 166.0 ± 7.2	Performance testing protocol (5 × 12 minutes with 1 minute of recovery: all-out running sprints, agility, precision ball kicking), heart rate, blood lactate and RPE, diet with 7 g.kg^−1.^day^−1^ CHO, 5 g of creatine monohydrate per day for 6 days	Body mass increased from 61.7 kg to 62.5 kg (ranged 0.3-1.5 kg) in players that received creatine. This group improved the performance in velocity and agility tasks, decreased heart rate and blood lactate concentration.
Clark et al. [26]	Dietary intake	US	N = 14 University pre-season age (years): 19.7 ± 07 weight (kg): 62.0 ± 4.8 body fat (%): 16.4 ± 2.4 height (cm): 165.8 ± 5.1 post-season age (years): 20.0 ± 0.9, weight (kg): 61.6 ± 4.7 body fat (%): 16.1 ± 2.8 height (cm): 165.8 ± 5.1	3-day food diary (pre-season and post season), body composition assessed by underwater weighing	No significant changes were noted across the season in body mass and fat mass percentage. During the pre-season daily energy (2251 kcal.day^−1^), CHO (5.2 g.day^−1^, protein (1.4 g.day^−1^) and fat intakes (29% total kcal) were significantly higher than post-season (daily energy: 1865 kcal.day^−1^, CHO: 4.3 g.day^−1^, protein: 0.96 g.day^−1^, fat: 13.3% total kcal). The intake of micronutrients (vitamin E, folate, copper, and magnesium) was not met. In periods of intense training, players met the energy and protein recommendations but failed to follow the guidelines for CHO and micronutrients.
Mullinix et al. [27]	Dietary intake	US	N = 18 Elite age (years): 19.2 ± 1.1 weight (kg): 59.7 ± 7.1 height (cm): 162.1 ± 12.6	3-day food diary	Participants consumed 2015 kcal.day^−1^ (282 g CHO (55%), 79 g protein (15%), 67 g fat (30%)). The intake of vitamin D and vitamin E did not follow the guidelines and intake of minerals was above the recommendations. Players ate 4-6 meals per day. More than 50% of participants used sports beverages and, approximately 39% consumed energy bars. Note 5.6% of the players used ephedra and fat burners.
Abood et al. [28]	Nutritional knowledge – education intervention program	US	N = 15 University age (years): 19.6 ± 1.0 weight (kg): 61.9 ± 5.8 height (cm): 167.4 ± 6.1	Questionnaires and 3-day food diary. The intervention focuses 1-hour educational sessions per week.	The intake of soccer players was, on average, 1969 kcal.day^−1^ at baseline (59% CHO, 24% fat, 13% protein). The intervention improved nutritional knowledge, self-efficacy and dietary changes.
Kang et al. [29]	Supplementation – iron	Korea	N = 11 Elite supplementation age (years): 22.6 ± 2.0 weight (kg): 57.9 ± 4.5 body fat (%): 24.1 ± 3.1 height (cm): 165.2 ± 6.0 N = 14 control age (years): 23.8 ± 2.8 weight (kg): 57.0 ± 4.9 body fat (%): 23.7 ± 3.1 height (cm): 163.9 ± 5.7	24-h recall. Body composition was estimated by skinfolds, biochemical parameters of blood and plasma. Experimental group was Supplemented with 40 mg per day.	Daily EI was comparable in both groups (supplemented: 2154 kcal.day^−1^; control: 2083 kcal.day^−1^). The intake in the group supplementing with iron was 78 g, 68 g and 312 g of protein, fat and CHO, respectively. Data for the control group was 66 g, 66 g, 292 g for protein, fat and CHO. Over the 4 weeks of the study, iron supplementation blood hemoglobin concentration remained constant in the supplementation group but decreased significantly in the control group. In addition, changes in mean cell volume and plasma ferritin increased significantly in the supplementation group.
Martin et al. [30]	Energy expenditure/ dietary intake	UK	N = 16 Elite age (years): 25.5 ± 3.9 weight (kg): 61.5 ± 5.3 body fat (%): 24.1 ± 3.1 height (m): 1.67 ± 0.08	7-day food diary and diary for physical activity levels.	Daily EI was 1904 kcal.day^−1^ while, daily EE was 2154 kcal.day^−1^. The daily relative EI was 30.9 kcal. kg^−1.^day^−1^. CHO and protein intake were 4.1 g. kg^−1^ and 1.2 g. kg^−1,^ respectively. Vitamin A (336.1 ± 195.0 mg) and iron (12.1 ± 6.0 mg) did not meet reference nutrient intakes. Mean fluid intake was 2466 ml.day^−1.^
Ali et al. [31]	Fluid intake	New Zealand	N = 10 National league age (years): 25.5 ± 5.2 weight (kg): 63.5 ± 5.7 height (m): 1.68 ± 0.05	Randomized-crossover trial. Intervention: players ingested water at a rate equivalent to 3 ml. kg^−1^ of body mass after every 15 minutes of exercise. In the control group, no fluid was ingested. Independent variables include urine sample, blood lactate and perceptual variables.	The ingestion of water reduces the hydration in soccer and also has impact on intestinal temperature, heart rate, blood lactate and RPE. Changes in performance were comparable in fluid and non-fluid ingestion.
Gibson et al. [32]	Energy expenditure/ dietary intake	Canada	N = 33 Junior national league age (years): 15.7 ± 0.7 weight (kg): 60.9 ± 8.2 height (cm): 163.8 ± 5.9 sum of skinfolds (mm): 103.1 ± 35.2	4-day food diary. EE used specific physical activity factors and energy activities constants according to the level’s activity (i.e. rest, training, match).	EI (2079 kcal.day^−1^) was significantly lower than EE (2546 kcal.day^−1^). Relative EI was, on average, 35 kcal.kg^−1^. CHO and protein intake were, on average, 5.0 g.day^−1^ and 1.4 g.day^−1^, respectively. Compared to recommend dietary intake, micronutrients recommendations were not attained for vitamin D, vitamin E, magnesium, phosphorus, vitamin A, vitamin B_12_ and zinc. Considering the recommendations for athletes, most of the players had insufficient levels of iron (89%) and 25-hydroxyvitamin D (50%).
Guzman et al. [33]	Supplementation – (DHA)	Spain	N = 24 National league age (years): 23.5 ± 5.2	Double-blind randomized trial. Experimental group: 3.5 g.day^−1^ of DHA; placebo: olive oil over 4 weeks. 24-hour over 7 days was used to determine energy and macronutrients intake. Multiple reactions time protocol tested the complex reaction efficiency considering visual and auditory stimuli.	EI for all players was 3050 kcal.day^−1^ (44% fat, 40% CHO, 16% protein). The supplementations with DHA had positive effects on complex reaction time and efficiency.
Astorino et al. [34]	Supplementation – Red Bull energy drink	US	N = 15 University age (years): 19.5 ± 1.1 weight (kg): height (cm): 168.4	Single-blind randomized crossover trial. 225 ml of Red Bull (110 kcal, 80 mg of caffeine, 1 g of taurine, 27 g of CHO) 1 hour pre-exercise. Performance was evaluated by 24 ‘*all out*’ sprints and the following parameters were collected: mean sprint time, heart rate and RPE. Onset of symptoms or side effects were also collected.	Comparable results were obtained for the Red Bull and placebo group regarding mean sprint time, heart rate and RPE. The effects of Reb Bull were considered negligible.
Gibson et al. [35]	Fluid intake	Canada	N = 34 National youth league age (years): 15.7 ± 0.7	Urine specific gravity (USG), sweat loss and sweat sodium concentration.	Before the practice, 45% of players were hypo hydrated. Sweat losses, expressed by changes in body weight, and sweat sodium concentration were, on average, 0.84% and 0.69 l, respectively. Fluid intake was moderate (64% ingested less than 250 ml). Fluid intake was not enough to fit sweat and sodium losses.
Gravina et al. [36]	Dietary intake	Spain	N = 28 National league age (years): 21.0 ± 6.0 weight (kg): 61.0 ± 8.4 body fat (%): 16.7 ± 3.2	8-day food diary, blood parameters.	EI was, on average, 2271 kcal.day^−1^ (CHO: 44.3%; protein: 15%; fat: 37%. The intake of fiber was 20 g.day^−1^. The relationship between fatty acids was examined considering two ratios: polyunsaturated/saturated fatty acids (PUFAs/SFAs) and polyunsaturated + monounsaturated/ saturated fatty acids (PUFAs +MUFAs/SFAs) and, both were below the recommendations. Athletes did not meet the recommendations of folic acid, vitamin D, iodine, magnesium and potassium. Briefly, it was noted a relationship between the nutritional intake muscle damage, oxidative stress, immunity and inflammation markers.
Reed et al. [37]	Energy expenditure/ dietary intake	US	N = 19 University age (years): 19.2 ± 0.3 weight (kg): 60.6 ± 1.4 height (cm): 165.6 ± 1.2	3-day food diary. Three methods to calculate EE: polar team software, polar FT4 heart rate monitor, metabolic equivalents. Body composition was assessed by DXA. Questionnaire were used to assess eating attitudes. A treadmill protocol and blood samples were also used.	EI was higher in pre-season (2794 kcal.day^−1^) compared to mid-season (2208 kcal.day^−1^) and post-season (2161 kcal.day^−1^). Exercise EE also decreased over the season (pre-season: 819 kcal.day^−1^; mid-season: 642 kcal.day^−1^; post-season: 159 kcal.day^−1^. Low energy availability was associated with body dissatisfaction and thinness. A reduced percentage of participants were at risk for low energy availability.
Lara et al. [38]	Supplementation – caffeine	Spain	N = 18 National league age (years): 21.0 ± 2.0 weight (kg): 57.8 ± 7.7 height (cm): 161.0 ± 6.0	Double blind randomized control trial. Players ingested 3 mg.day^−1^ caffeine. After one-week, participants ingested a drink without caffeine. Countermovement jumps and 7 ⅹ 30 speed test. 2 ⅹ 40 min soccer match with GPS/HR device. Urine sample. Questionnaires about match sensations, sleep quality, nervousness, gastrointestinal problems.	The ingestion of caffeine increased jump height and peak speed during the sprint test compared to placebo group. Match activities also changed significantly when players ingested caffeine. The total distance covered, number of sprints, and running distance covered at velocities >18 km.hour^−1^ were higher in the caffeine group compared to placebo.
Reed et al. [39]	Energy expenditure/ dietary intake	US	N = 19 University age (years): 19.0 ± 1.0	3-day food diary. Three methods to calculate energy expenditure: polar team software, polar FT4 heart rate monitor, metabolic equivalents. Body composition was assessed by DXA. A treadmill protocol was used.	Players grouped as lower energy availability did not met the recommendations for CHO and protein. Percentage of kilocalories derived from food, sport drinks, bars/gels/beans were compared in players with low and high energy availability.
Gonçalves et al. [40]	Dietary intake	Brazil	N = 7 National league age (years): 20.1 ± 3.3 weight (kg): 57.6 ± 8.6 body fat (%):23.7 ± 4.9 height (m): 1.65 ± 0.06	Body composition was assessed by bioimpedance. 24-h recall (3 days).	The daily EI was 2386 kcal, 1940 kcal, 2282 kcal on training days, match day and post-match. When the values were expressed per kg of body mass, the respective values were 45.3, 34.2 ad 41.8. Relative intake of CHO was 5.9 g.kg^−1^, 4.3 g.kg^−1^ ad 5.3 g.kg^−1^ were on training days, match day and post-match. The relative protein intake was 1.9 g.kg^−1^ on training day, 1.5 g.kg^−1^ on match day, 1.9 g.kg^−1^ post-match. Percentage of fat intake was approximately 30%. It was also noted an insufficient intake of vitamins and minerals. Glycemic load was high during the three days of the study.
Gonzalez-Neira et al. [41]	Dietary intake	Spain	N = 17 National league age (years): 22.1 ± 4.6 weight (kg): 61.5 ± 7.9 body fat (%): 24.5 ± 5.6 height (cm): 165.2 ± 6.8	Body composition was assessed by bioimpedance. 7-food diary and KIDMED. EE was estimated using an equation to predict REE and multiplied it by a physical activity level. Protocol Course-Navette was used to estimate maximal oxygen uptake. RPE.	EI was, on average, 1901 kcal.day^−1^ (CHO: 40%; protein: 16%; fat: 42%). A negligible percentage of athletes (i.e. 5.9%) showed an optimal adherence to the Mediterranean diet.
Mara et al. [42]	Energy expenditure/ dietary intake	Australia	N = 8 Elite age (years): 23-30 weight (kg): 65.1 ± 5.9 body fat (%): 23.2 ± 6.2 height (cm): 172.9 ± 5.5	EE, sport EE, training and match activities were obtained using SenseWear Mini Armbands (SWAs) and GPS.	Using the SWAs, the daily EE was 2933 kcal in match, 2793 kcal in training days and 2272 kcal in rest days. Exercise EE was 620 kcal in match day and 606 in training days. Differences between SWAs and GPS were noted.
Castro-Sepulvedaet al. [43]	Fluid balance	Chile	N = 17 Elite age (years): 21.5 ± 3.0 weight (kg): 62.0 ± 6.0 height (cm): 165.0 ± 9.0	Questionnaire was used to examine the importance of hydration for each player. urine specific gravity allowed to examine hydration status prior to training sessions, friendly or official matches.	Percentages of players classified as severely dehydrated, dehydrated, mildly dehydrated and, euhydrated were, approximately 47%, 33%, 18% and, 2%, respectively. Values of urine specific gravity tended to be higher prior to official matches in comparison to friendly matches.
Santos et al. [44]	Dietary intake	Brazil	N = 21 Elite age (years): 20.9 ± 4.5 weight (kg): 56.9 ± 6.3 body fat (%): 14.6 ± 2.3	24-h recall (3 days). EE was calculated using the metabolic equivalent task for each type of physical activity. Skinfolds were used to determined body fat percentage. Healthy eating index was used to evaluate diet quality.	EE was 2306 kcal.day^−1^. The intake of CHO was 5.5 g.kg^−1.^day^−1^ while the ingestion of protein was 2.0 g.kg^−1.^day^−1^. The percentage of saturated fat was 10.1% while, fiber and sodium were 16.5 g and 2.3 g, respectively. Total EE was, on average, 2701 kcal.day^−1^ while EI was 2306 kcal.day^−1^. The ingestion of CHO, protein and fat were 5.5 g.kg^−1^, 2.0 g.kg^−1^, respectively. The percentage of fat was 26.3%. The healthy eating index was, on average, 54.6 which may indicate that diet quality should be improved.
Prather et al. [45]	Female athlete triad	US	N = 320 University age (years): 10–30	Questionnaires to collect age, height, weight, age at menarche, menstrual function, history of eating disorders, musculoskeletal injuries and eating disorders. Eating attitudes test questionnaire.	Overall, players were at low risk of eating disorders. Players that reported ≥ 10 points in the eating attitude test questionnaire had menstrual dysfunction. 19 athletes reported stress fractures in lower extremities. Only two athletes that reported ≥ 10 points also had stress fractures in lower limbs.
Přibyslavská et al. [46]	Supplementation – CHO during exercise	US	N = 11 University age (years): 20.0 ± 1.0 weight (kg): 59.6 ± 3.3 height (cm): 164.0 ± 6.0	Double blind crossover design. Mouth rinsing protocol (10–15 seconds) included a CHO or placebo beverages. The intervention protocol was designed with 3 vs. 3 high intensity exercises and performance tasks (vertical jump, shuttle run sprint).	No significant differences between groups were found in performance, RPE and thirst sensation which did not support the use of mouth rinsing protocols on anaerobic tasks.
Ramírez-Campilloet al. [47]	Supplementation – creatine	NR	N = 30 Not professional age (years): 19–28	Double blind randomized placebo control trial. Three groups were considered: plyometric; plyometric + creatine, placebo. The intervention elapsed 6-weeks. Creatine was administrated in two different phases: 20 g.day^−1^ during one week and then, a single dosage of 5 g.day^−1^ during five weeks. Dietary intake was recorded using 24-h food record questionnaire in three days. Physical tests were combined drop jump reactive indexes, peak jump power, unilateral drop jump reactive strength index, change of direction test and 20-m multistage stage shuttle run test.	The group that combined supplementation (i.e. creatine) and plyometric training tend to improve the performance on jumping and sprint tests. The daily energy and nutritional intake were reported before and after the intervention for each group.
Chapelle et al. [48]	Fluid intake	Belgium	N = 18 Elite age (years): 17.6 ± 0.4 weight (kg): 61.4 ± 5.2 height (cm): 168.0 ± 4.3	Hydration status was measured by urine specific gravity during an official tournament.	At the beginning of tournament, a considerable percentage of players were hypo hydrated (44–78%). Considering that players received information about the hydration status, the percentage of players classified as euhydrated was 89%.
Hosseinzadeh et al. [49]	Dietary intake	Iran	N = 8 National youth league age (years): 13.1 ± 0.6 weight (kg): 53.3 ± 11.3 height (cm): 160.3 ± 4.5 body fat (%): 20.6 ± 3.2	Body fat percentage was measured using a bioelectrical impedance. Daily energy and nutritional intake were estimated by 24-h recall questionnaire applied for three days.	Daily EI was, on average, 3122 kcal.day^−1^. The nutritional intake was expressed in g – mean values: 445 g of CHO; 86 g of protein, 110 g of fat.
Manore et al. [50]	Nutrition knowledge	US	N = 297 Youth competition age (years): 15.2 ± 1.1 weight (kg): 59.5 ± 10.4 height (cm): 162.6 ± 6.7	Two questionnaires: demographic and health questionnaire and sport nutritional knowledge questionnaire.	Approximately 96% of female participants recognized the importance of nutrition to performance. Less than 50% of females reported that they ate the breakfast.
Mattausch et al. [51]	Fluid intake	US	N = 10 University age (years): 20.4 ± 1.4 weight (kg): 64.0 ± 9.0 height (cm): 166.0 ± 7.0	Intervention about hydration information. Changes in body weight, urine specific gravity, fluid intake and urine color.	A substantial proportion of the players start the exercise dehydrated. A long term and individualized intervention (4 weeks) allowed to the players being hydrated before the competition period.
Braun et al. [52]	Energy expenditure/ dietary intake	Germany	N = 56 National youth league age (years): 14.8 ± 0.7 weight (kg): 56.8 ± 6.1 height (cm): 166.0 ± 5.1 body fat (%): 17.2 ± 3.9	Daily EE and EI estimated using a 7-day records. Blood samples.	Daily EE (2403 kcal.day^−1^) and EI (2262 kcal.day^−1^) were comparable across one week. Consequently, a negative energy balance of – 141 kcal was obtained and a mean energy availability of 30 kcal.day^−1.^kg.LBM^−1^ was estimated. The relative intake of CHO, protein and fat was 5.4 g.kg^−1^, 1.4 g.kg^−1^ and 1.4 g.kg^−1^, respectively. Regarding micronutrients intake, the recommendations for Vitamin D intake was not met by any player. In addition, more 50% of the sample did not meet the recommendations for vitamin B_12_, folate, calcium and iron. Blood samples classified 17% of players as iron depleted and 38% of participants presented an inadequate status of vitamin D.
Cherian et al. [53]	Energy expenditure/ dietary intake	India	N = 19 National youth league age (years): 12.2 ± 1.83 weight (kg): 45.1 ± 6.58 height (cm): 153.6 ± 4.64 body fat (%): 23.8 ± 3.46	Body composition was estimated by anthropometry. Daily EI was evaluated using the direct weighment method for 3 days and confirmed to 24-hour dietary intake pattern while, daily EE was estimated using a portable metabolic analyzer and records.	Daily EE (2442 kcal.day^−1^) was related with daily EI (2243 kcal.day^−1^). An excessive intake of CHO was noted (7.9 g.kg^−1^) while the ingestion of fat (25.6%) and protein (1.5 g.kg^−1^) are, on average, adequate. The timing and quantities of macronutrients in pre-training, in-training and post-training did not follow the recommendations. Results about micronutrients noted that iron, zinc, vitamin A, vitamin C and calcium intake are lower than recommendations. In contrast, folic acid and vitamin D were in accordance with the recommended.
Cherian et al. [54]	Resting energy expenditure	India	N = 19 National youth league age (years): 12.2 ± 1.83 weight (kg): 45.1 ± 6.58 height (cm): 153.6 ± 4.64 body fat (%): 23.8 ± 3.46	The two compartments of body mass (i.e. fat mass and fat-free mass) were estimated with anthropometry. REE was measured and predicted using different equations.	Measured REE was, on average, 1135 kcal.day^−1^. Significant differences were noted for predicted REE obtained by Cunningham and Henry equations as well as De Lorenzo, Wong and ten Haaf estimations. Absolute values of REE tend to increase with age however, these differences are trivial when REE was normalized for weight or fat-free mass.
Dobrowolski et al. [55]	Dietary intake	Poland	N = 41 National league age (years): 21.0 ± 5.0 weight (kg): 62.2 ± 6.7	Daily EI was recorded using 3-day food diary and an armband device estimated the EE, physical activity level and metabolic equivalent task.	The average EI was 1476 kcal.day^−1^. Relative values for energy, CHO, protein and fat were 24.3 kcal. kg^−1.^day^−1^, 3.3 g. kg^−1.^day^−1^, 1.2 g. kg^−1.^day^−1^, 0.78 g. kg^−1.^day^−1^. More than 50% of the players did not met the recommendations for macronutrient intake. The same trend was reported in micronutrients (potassium, calcium, magnesium, iodine, vitamin B_1_, folate).
Pilis et al. [56]	Dietary intake	Poland	N = 15 Different competitive levels age (years): 21.3 ± 2.7 weight (kg): 60.0 ± 8.9 height (cm): 165.5 ± 6.0	Body composition was assessed using a bio-electrical impedance. EI was recorded by a 3-day food record.	EI was, on average, 1714 kcal.day^−1^ and daily relative EI was 28.59 kcal. kg^−1^. The relative intake of protein was 3.59 g. kg^−1^. The percentage of intake in fat was, approximately, 29%.
Yli-Piipari et al. [57]	Energy expenditure/ dietary intake	US	N = 13 University age (years): 19.9 ± 1.4 weight (kg): 59.9 ± 4.9 height (cm): 166.6 ± 4.9	EE was estimated using accelerometers and records. Dietary EI was studied using a 4-day food diary.	Daily EE was, on average, 2485 kcal.day^−1^ and EI was 1894 kcal.day^−1^. A deficit energy score was obtained (i.e. – 590 kcal.day^−1^). Macronutrients intake was 4.21 g.kg^−1.^day^−1^, 1.56 g.kg^−1.^day^−1^ and 1.31 g.kg^−1^.day^−1^ for CHO, protein, and fat, respectively.
Behrens et al. [58]	Energy expenditure/ dietary intake	US	N = 20 University age (years): 18.9 ± 1.0 weight (kg): 59.0 ± 7.9 height (cm): 163.9 ± 6.9 body fat (%): 19.8 ± 5.3	Body composition was estimated with bioelectrical impedance. 3-day records were used to estimate EE and EI.	EI was, on average, 2112 kcal.day^−1^ and EE was 2240 kcal.day^−1^. Across the day players were approximately 7 hours in energy deficit, 14 hours in energy balance and, 3 hours on positive energy balance. Energy balance and positive energy balance was negatively associated with fat mass percentage.
Dobrowolski et al. [59]	Iron intake	Poland	N = 38 National league age (years): 21.0 ± 5.0 weight (kg): 59.2 (median) height (cm): 167.0 ± 5.0	IRONIC-FFQ as well as 3-day food diary were used to estimate the intake of iron.	The intake of iron was considered adequate using both methods (IRON-FFQ was 8.06 mg; 3-day food diary record was 8.8 mg). In addition, IRON-FFQ can be used to estimate iron intake.
Dobrowolski et al. [60]	Energy expenditure/ dietary intake	Poland	N = 31 National league age (years): 21.5 ± 4.9 weight (kg): 58.0 (median) height (cm): 166 ± 5	Fat-free mass was measured with a bioelectrical impedance. Daily EI was assessed using a 4-day food diary. Daily and exercise EE were measured with an armband.	Daily EE was 2703 kcal.day^−1^ and, daily EI was 1548 kcal.day^−1^. Energy availability was, on average, 25 kcal.kg_FFM_^−1.^day^−1^. Of note, 20 players have lower values of energy availability.
Magee et al. [61]	Dietary intake	US	N = 18 University age (years): 19.2 ± 1.1 weight (kg): 65.3 ± 7.9 height (m): 1.67 ± 0.1 body fat (%): 24.9 ± 5.6	Air displacement plethysmography measured fat-mass and fat-free mass. A 4-day food diary estimated daily EI and, a wearable device measured daily EE. Two questionnaires about low energy availability and nutritional knowledge were applied.	Daily EI was 1931 kcal.day^−1^and, relative values for CHO, protein and fat were 3.7 g.kg^−1^, 1.2 g.kg^−1^, 1.1 g.kg^−1^, respectively. The mean energy availability was 27.5 kcal.kgFFM^−1^.day^−1^. A substantial percentage of players (66%) were at low energy availability. These players had a poor nutritional knowledge. The questionnaire to identify players at low energy availability has limitations.
Ribeiro et al. [62]	Supplementation – beta-alanine	Brazil	N = 24 Elite age (years): 18 ± 1 weight (kg): 62.7 ± 7.4 height (cm): 1.67 ± 0.07	Double-blind placebo study. Physical tests: YOYO IR1, repeated ability sprint test and, 20-m sprint test. Participants received 6.4 g.day^−1^of beta-alanine or maltodextrin.	The participation in 3-week of intense training program prior to an important competition did not improve high-intensity exercise. Note, the influence of beta-alanine did not have impact on performance.
Wang et al. [63]	Fluid balance	US	N = 14 age (years): 19.0 ± 1.0 weight (kg): 68.5 ± 9.0 height (cm): 168.4 ± 9.0	Body mass change, sweat rate, urine, sweat electrolyte concentrations, fluid intake.	A significant number of players (54%) were hypo hydrated prior to practice (urine specific gravity > 1.020). Differences by playing position. Fluid intake was higher in defenders compared to forwards, midfielders and forwards. Lower changes on body weight were also noted in defenders. Sweat rates were elevated on forwards.
Aboott et al. [64]	Eating disorders	UK	N = 70 National league	Two questionnaires: clinical perfectionism and eating attitudes test.	The prevalence of eating disorders in female soccer players was lower than in the control group. Perfectionism was a significant predictor of disorder eating.
Clarke et al. [65]	Fluid balance	US	N = 16 University age (years): 20.4 ± 0.8 weight (kg): 65.3 ± 12 height (cm): 163.6 ± 6.9	Urine specific gravity was analyzed in the morning and afternoon/post-practice.	The results indicated that large proportion of players awoke and start practice or matches in a hypo hydrated state.
Łuszczki et al. [66]	Resting energy expenditure	Poland	N = 35 Youth competition age (years):14.5 ± 1.8 weight (kg):54.0 ± 8.0 height (cm):163.7 ± 9.2	REE measured by indirect calorimetry, body composition was examined using DXA, arterial pressure.	REE was related with muscle mass and height.
Łuszczki et al. [67]	Dietary intake	Poland	N = 34 Youth competition age (years): 15.4 ± 4.2 weight (kg): 55.9 ± 5.5 height (cm): 166.4 ± 5.7	REE measured by indirect calorimetry, body composition was examined using DXA, EI was obtained by a 24-h food recall and, physical activity level was set at 1.8. A Low energy availability questionnaire was applied.	The mean EI was 1872 kcal.day^−1^. More than 50% of participants was at risk of low energy availability according to the questionnaire applied. Participants at risk of low energy availability presented a lower EI (1773 kcal) compared to participants classified as at-risk (2054 kcal). The questionnaire is a useful tool to identify participants at low energy availability.
Moss et al. [68]	Energy expenditure/ dietary intake	UK	N = 30 Elite age (years): 23.7 ± 3.4 weight (kg): 63.7 ± 7.0 hight (cm): 1.69 ± 0.1	Body composition was measured with DXA. EI were measured during a 5-day food diary. RMR was measured using indirect calorimetry and EE during exercise was estimated by metabolic equivalent task (MET). Blood samples allowed to collect data about micronutrients and biochemical markers. Two questionnaires were used: Low Energy Availability in Females Questionnaire and Eating Pathology.	Energy availability was low in 62% of participants and it tends to be higher in resting days compared to training sessions and competition. Meantime, mean values for EI, CHO and protein were 2124 kcal, 3.3 g.kg^−1^ and 1.8 g.kg^−1^, respectively. 23% of participants were at risk of low energy availability.
Campenhout et al. [69]	Fluid intake	Belgium	N = 37 National league intervention group (n = 22) age (years): 19.8 ± 3.0 weight (kg): 62.7 ± 7.6 control group (n = 15) age (years): 22.8 ± 4.0 weight (kg): 62.2 ± 7.1	The duration of intervention was 2 weeks (two training sessions and one competition). The individual and instructional tailored intervention occurred between the weeks. The intervention group received information about being hydrated based on urine specific gravity.	The intervention group decreased the urine specific gravity. In contrast, control group increased the values of urine specific gravity. Individual interventions related to hydration are needed to improve hydration status.
Wynne et al. [70]	CHO before exercise	US	N = 15 University age (years): 19.6 ± 1.3 weight (kg): 67.5 ± 12.9 height (cm): 165.9 ± 9.1	Randomized crossover design – diet high CHO and diet with mixed-macronutrients meal consumed prior 4 hours before soccer games. Global position system data, heart rate, ratings of fatigue and exertion, gastrointestinal ratings, ratings of hunger, fullness and satiety.	No significant differences were obtained for both conditions which may indicate that a mixed meal can be consumed 4 hours prior to soccer competition.
Dasa et al. [71]	Energy expenditure/ dietary intake	Norway	N = 13 National league age (years): 20.5 ± 4.3 weight (kg): 168.4 ± 5.1 height (cm): 64.1 ± 5.3	Three commonly devices (GPS), Apex, StatSport, Newry, Northern Ireland, UK; GPS, Vector, Catapult innovations, Melbourne, Australia; IMU, PlayermakerTM, Tel Aviv, (Israel) were compared against indirect calorimetry on EE. Blood lactate was also measured.	Compared to the indirect calorimetry, the three tracking devices tend to underestimate the caloric expenditure during intermittent bouts of exercise. After corrected for excess post-oxygen uptake differences between devices were reduced. The error also shows a specifically pattern – devices overestimated EE at lower levels of caloric expenditure and underestimated EE at higher levels of caloric expenditure.
Günalan et al. [72]	Supplementation – prevalence	Turkey	N = 38 National league age (years): 24.3 ± 4.8 weight (kg): 57.5 ± 5.6 height (cm): 167.3 ± 5.9	A questionnaire was used to assess the prevalence and patterns of supplements used by athletes. Supplements were categorized into the categories defined by the Australian Institute of Sports.	73.7% of female players ingested supplements and tend to purchase from the pharmacy. Female participants also tend to intake supplements during the competition. The types of supplements ingested by females were sport drinks (55.3%), sport bars (26.3%), vitamin D (36.8%), zinc (21.1%), caffeine (23.7%), vitamin C (54.4%), omega-3 fatty acids (10.5%), magnesium (47.7%), green tea (10.5%).
Leão et al. [73]	Energy expenditure/ dietary intake	Portugal	N = 14 National league age (years): 22.5 ± 4.4 weight (kg): 57.2 ± 8.6 height (cm): 164.0 ± 6.0 body fat (%): 18.3 ± 2.5	Body composition was estimated using an anthropometric equation. 7-day food diary were filled to estimate EI. External and internal load on match and training days were obtained from GPS and RPE, respectively.	The mean EI was 1764 kcal.day^−1^. Daily EI, normalized for fat-free mass was, on average, 38.9 kcal.day^−1.^kg_FFM_^−1^. EI and CHO tended to be lower on days preceding the competition than during training days. In general, no associations were found between soccer load and nutritional intake.
McKinlay et al. [74]	Effects of Greek yogurt	Canada	N = 13 Youth league age (years): 14.3 ± 1.3 weight (kg): 59.1 ± 2.1 body fat (%): 22.2 ± 1.8	Double-blind randomized crossover trial. The intervention consists in 5 consecutive days of intense soccer training whereby participants consumed 160 of Greek yogurt or 30 g of isoenergetic CHO drink post-training, 1 h prior to bedtime and, between breakfast and lunch. 24 hour food-records and food frequency questionnaire were used to assess nutritional intake. A battery test was performed preceding each trial. Blood samples were collected and analyzed.	Habitual EI was, on average, 1622 kcal.day^−1^. Relative intake of CHO, proteins and fats was 3.3 g.kg^−1^, 1.1 g.kg^−1^, 1.1 g.kg^−1^, respectively. There was no intervention effect on performance. Regarding the biomarkers analyzed it was noted an increase of inter-leucine 10 when Greek yogurt was ingested. However, negligible changes were verified for inter leucine 6, TNF α and creatine-kinase. In brief, the positive impact of Greek yogurt on performance and recovery was not evident.
Morehen et al. [75]	Energy expenditure/ dietary intake	UK	N = 24 Elite height (cm): 168.1 ± 5.9 weight (kg): 62.1 ± 4.7 body fat (%): 11.8 ± 2.7 (N = 18)	Body composition was evaluated using DXA and resting metabolic rate was predicted. EE was measured by doubly labeled water method during 4 or 12 days. 4-day weight food inventory.	Daily EE was comparable considering 4 (2753 kcal) or 12 days (2693 kcal) of analysis. Daily EI was, on average, 1923 kcal. Of note, 88% of players were categorized with low energy availability (< 30 kcal.kg_FFM_^−1^). The intake of CHO was, on average, 3.3 g.kg^−1.^day^−1^. Energetic requirements are comparable between male and female soccer participants.
Oliveira et al. [76]	Supplementation – prevalence	Portugal	N = 103 National league age (years): 24.0 ± 5.0 height (cm): 169.0 ± 8.0 weight (kg): 63.4 ± 7.0	Questionnaire with three domains: (1) demographic and anthropometric characteristics, (2) exercise practice over the previous 12 months, (3) use of supplements in the previous 12 months.	The majority of participants (i.e. 82%) consumed at least once over the last 12 months. The preferable supplements were vitamin D, omega-3 fatty acids and protein. Health and performance reasons were commonly reported to justify the usage of supplements.
Tarnoskwi et al. [77]	Fluid balance and CHO intake during exercise	Spain	N = 19 National league height (cm): 169.4 ± 5.9 weight (kg): 61.0 ± 5.4 body fat (%): 21.1 ± 4.1	Players were examined in training and match days. Fluid balance was measured by urine-specificity gravity while, thirst was evaluated using a scale (1 represents ‘not thirsty at all’ and 7 indicates ‘very, very thirsty’). Regional absorbent sweat patches were positioned on the players to measure sweat rate and sodium losses. During exercise, water, CHO-electrolyte beverages, bananas and sport gels were available.	The mean value of urine specific gravity was higher prior to the training session than the match day. Changes in body mass are more pronounced on match day (−1.12%) than training days (+0.29%). Sweat rates and sodium losses, expressed in mg.h^−1^ were greater during the match compared to training. The ingestion of fluid and CHO was comparable in training and match days. The intake of CHO was 2.0 g.h^−1^ and 0.9 g.h^−1^ during training and match days, respectively. Electrolyte beverages were the unique source of CHO.
McHaffie et al. [78]	Dietary intake	UK	N = 12 National league	Semi-structured interviews.	Female players are not aware about the energetic recommendations. Under-fueling is commonly reported in female athletes and this aspect seems to be caused by the misunderstandings about the impact of CHO intake.
Essman et al. [79]	Omega 3 intake	US	N = 31 University height (cm): 169.8 ± 6.1	Brief Omega 3 food frequency questionnaire, erythrocyte analyses.	The questionnaire results were associated with the erythrocyte analysis of omega 3 fatty acids.
Sebastiá-Rico et al. [80]	Dietary intake	Spain	N = 105 National league	Food frequency questionnaire.	Female players ingested more red meat than males. Fruits, vegetables and red meat are the most ingested food by females. Most of the players did not ingest alcoholic beverages.
Paludo et al. [81]	Energy expenditure/ dietary intake, eating behavior	Cyprus	N = 20 Youth competition age (years): 14.6 ± 1.4 weight (kg): 54.3 ± 7.6 height (cm): 145.0 ± 9.0	Low energy availability questionnaire, orthorexia nervosa questionnaire, physical tests.	The prevalence of low energy availability was 26.3%. Low energy availability was associated with higher values of orthorexia nervosa questionnaire. However, these scores are not related with performance decrements during the pre-season.
McHaffie et al. [82]	Energy expenditure/ dietary intake	UK	N = 23 National league age (years): 17.9 ± 0.5 weight (kg): 61.6 ± 6.1 body fat (%): 25.7 ± 3.3 height (cm): 168.0 ± 5.0	Bioeletrical impedance, GPS was used to quantify training and match load, self-report physical activity, 10-day food diary.	On match day two (4.8 ± 1.4 g.kg^−1^) and the days before match one (4.1 ± 0.8 g.kg^−1^) and two (4.8 ± 1.2 g.kg^−1^) the intake of CHO was greater in comparison to other days (< 4 g.kg^−1^). The mean daily energy availability during 10 days was 34 ± 12 kcal.kg_FFM_^−1.^day^−1^. 34% of players had low energy availability. The adjustment of under-reporting may indicate that low energy availability is over-reported although, players did not follow the nutritional guidelines for match and training schedules.
Kwon et al. [83]	Dietary intake (protein)	US	N = 23 University age (years): 19.4 ± 1.5 weight (kg): 63.5 ± 5.5 height (cm): 167.0 ± 5.4	3-day food diaries.	The amount protein (81%) ingested came from breakfast, lunch and dinner. The amounts of protein at breakfast or lunch are not attained.
Dobrowolski et al. [84]	Energy expenditure/ dietary intake	Poland	N = 7 Elite age (years): 23.4 ± 6.6 weight (kg): 63.5 ± 7.8 fat-free mass (kg): 46.0 ± 4.4 height (cm): 168.5 ± 5.8	Body size, SenseWear Pro3 Armband device was used to measure EE, biompedance was used to measure body composition.	The EE was substantially higher on match hour (452 ± 55 kcal.hour^−1^) than training period (353 ± 28 kcal.hour^−1^). Differences were maintained when the values were normalized for fat-free mass.
Dasa et al. [85]	Energy expenditure/ dietary intake	Norway	N = 51 Elite age (years): 22.0 ± 4.0 weight (kg): 63.9 ± 6.6 fat-free mass (kg): 49.3 ± 4.9 height (cm): 169.0 ± 7.0	Body composition was measured using DXA, training and match load were monitored with GPS. EI was assessed with 24-hour diet recalls for 3 days. Daily EE was examined with doubly labeled water.	Mean EE was 2918 ± 322 kcal.day^−1^ whilst the EI 2274 ± 450 kcal.day^−1^. CHO intake was below the recommendations (4.5 ± 1.9 g.kg^−1^). The prevalence of low energy availability was 36% and 23% on match and training days, respectively.
Choi et al. [86]	Supplementation – curcumin	Japan	N = 6 Elite age (years): 20.0 ± 2.0 weight (kg): 58.1 ± 6.3 height (cm): 162.1 ± 8.7	Single-blind, placebo-controlled, nonrandomized, crossover design: two different conditions during 14 days – placebo and curcumin supplementation separated by 7 days of wash-out. Resting blood pressure, biochemical parameters were measured.	Differences between conditions were negligible for blood pressure, heart rate and muscle damage parameters markers (CK, serum myoglobin, AST, LDH).
Atanasio et al. [87]	Effects of a nutritional program	Spain	N = 19 National league Controlled diet group age (years): 19.0 ± 1.4 weight (kg): 63.6 ± 10.0 height (cm): 164.0 ± 2.0 Exchange diet group age (years): 22.5 ± 4.8 weight (kg): 61.3 ± 7.0 height (cm): 164.0 ± 0.1	Randomized controlled trial with two different approaches during 12-weeks: controlled-diet group (i.e. defined menus) and exchange-diet (i.e. athletes designed own menus with an exchanged list). Dietary intake using 7-day food records, nutritional knowledge using the General Nutrition Knowledge Questionnaire. Skinfold thickness was measured, running speed and jumping performance were evaluated and blood samples were collected.	To improve nutritional the exchange diet seems to be a good strategy. Control diet may help to normalize hemoglobin concentration and prevent anemia. Apparently, skinfold thickness was reduced in both type of diets. Improvements were not noted in soccer-specific skills.
Sebastiá-Rico et al. [88]	Fluid balance	Spain	N = 11 National league age (years): 22.6 ± 3.5 height (cm): 165.0 ± 5.0 weight (kg): 61.6 ± 8.7	Percentage of weight lost, sweat rate, urine specific gravity measured using a digital refractometer, urine color.	Weight loss, sweat rate, fluid intake was higher during the summer in comparison to winter. Fluid balance was affected by sex, climatic conditions and competitive level (values tend to be higher in male soccer players).
Sebastiá-Rico et al. [89]	Supplementation – prevalence	Spain	N = 28 National league age (years): 22.0 ± 3.5	Sport supplement questionnaire with 28 questions aggregating three main domains: information, sports practice, nutrition and supplementation.	Sports supplements tend were consumed on training and extra-training days. Sports drinks were the most frequent supplement ingested by female soccer players (56%), followed by sports bars (50%) and whey protein (48.6%). Creatine and caffeine were consumed by 60% and 47.1% of female players, respectively.
Petri et al. [90]	Effects of a nutritional program	Italy	N = 44 Elite age (years): 27.0 ± 5.0 height (m): 1.69 ± 0.05 weight (kg): 62.2 ± 5.7	Examine the effects of a nutritional program following specific criteria: food first approach, individualized and personalized supplementation, mandatory main meals at the club context and, in parallel, an individual follow-up. Body composition was measured using bioimpedance and skinfolds.	Changes in body composition were noted in female players. More precisely, across the competitive seasons female players tend to decrease the levels of fat mass.
Molina-López et al. [91]	Supplementation– prevalence	Spain	N = 126 National league age (years): 24.6 ± 4.2 height (cm): 165.0 ± 6.0 weight (kg): 60.6 ± 6.7	Two different questionnaires were used: Nutritional Supplement and Consumption Questionnaire and Athlete Food Choice Questionnaire.	84% of participants ingested supplements to improve sports performance or maintain health. The most prevalent supplements ingested were whey protein (30%), sport drinks (29%), creatine (29%), sport bars (28%) and caffeine (28%). The ingestion of sport supplements was associated with food choices.
Coombes et al. [92]	Energy expenditure/ dietary intake	New Zeeland	N = 22 National league age (years): 20.8 ± 3.5	Five different questionnaires were used: low energy availability risk questionnaire, eating disorder examination questionnaire, athlete sleep score questionnaire, Abridged Sport Nutrition Questionnaire, profile of mood states questionnaire.	More than 50% of participants were at risk of low energy availability. Menstrual disturbances and mood states questionnaires indexes were associated the of low energy availability.
Brinkmans et al. [93]	Energy expenditure/ dietary intake	Netherlands	N = 15 National league age (years): 22.9 ± 4.5 weight (kg): 61.7 ± 3.3 body fat (%): 19.1 ± 3.0 height (cm): 168.8 ± 5.8	Body composition was measured with DXA. Doubly labeled was used to measure total daily EE expenditure. Local sensors allowed to obtain training and match load whilst, accelerometers were used to estimate physical activity. Nutritional intake was estimated by four unannounced 24-h dietary recalls.	Total daily EE was 2882 ± 278 kcal.day^−1^ whilst the total EI was, on average, 2344 kcal. CHO intake varied according competitive period (rest day: 3.2 ± 0.7 g.kg.day^−1^, training: 4.4 ± 1.1 g.kg.day^−1,^ match: 5.3 ± 1.9 g.kg.day^−1^). The average intake of protein was 1.9 ± 1.4 g.kg.day^−1^.

DXA (dual-energy X-ray absorptiometry); REE (resting energy expenditure); EE (energy expenditure); EI (energy intake); RPE (rate of perceived exertion); CHO (carbohydrates); DHA (docosahexaenoic acid); PUFA (polyunsaturated fatty acids); MUFA (monounsaturated fatty acids); SFA (saturated fatty acids).

The most common topics from the included studies were energy expenditure and nutritional intake [22,25,26,31,35,36,38–41,43,48,50–56,58,59,64–66,69,71,73,76,78–83,90,91] and fluid balance [30,34,42,47,49,61,63,67,70,75,86] The prevalence of supplement use was also documented in female soccer players [72,76,89,91]. The effectiveness of creatine was examined in three studies [24,25,47]. The effects of iron [29], docosahexaenoic acid [33], energy drink [34], caffeine [38], beta-alanine [62], and curmumin [86] were also included. Two studies documented the effects of CHO intake before [70] and during exercise [46]. Nutritional knowledge was reported in two studies of US female soccer athletes [28,50] and two studies investigated the impact of a nutritional program on body composition [87,90]. Three studies used questionnaires to investigate variables associated with the Female Athlete Triad [45], the prevalence of eating disorders [64], iron intake [59], and the intake of omega 3 [79]. A separate study tested the effects of Greek yogurt on performance and recovery biomarkers [74].

Energy intake was commonly assessed by using the food weight method [53,75], 8-day [36], 7-day [22,30,41,52,73], 5-day [68], 4-day [32,60,61] or 3-day food diaries [26,27,37,39,56,58] and 24-recall method [40,44]. Total or exercise energy expenditure using exercise diaries [22,30,52,58], equivalent metabolic tasks or physical activity level coefficients [32,37,39,41,68], portable instruments [37,39,42,53,55,60,61,71] and doubly labeled water over 4 and 12 days [75]. Studies also described the hydration status before, during or after training and matches [23,35,43,48,63,65,77].

### 3.3 Results of individual sources of evidence

Allowing for variation in methods used for daily energy intake and the level of participants, the caloric intake (kcal.day^−1^) was widely variable across studies of adult and youth female soccer players (Table 2). In adult players, daily energy intake ranged from 1476 kcal in professional Polish soccer players [55] to 3050 kcal in Spanish Super League players [33]. An average intake of ~2060 kcal was obtained for 30 studies that included adult female soccer players. The energy intake varied from 1622 kcal.day^−1^ in youth Canadian players [74] to 3122 kcal.day^−1^ in youth Iranian players [49]. Different methods were used to obtain daily energy expenditure. One study measured energy expenditure by doubly labeled water; with energy expenditure identified as 2963 kcal.day^−1^ [75]. Three studies characterized energy intake at different phases within the season in adult participants [26,37,40]. The daily energy intake was substantially higher in pre-season (2290 kcal.day^−1^) versus post-season (1865 kcal.day^−1^) in US soccer players [26]. Comparable findings were noted in another study conducted with adult American players [37], with the intake during pre-season (2794 kcal.day^−1^) higher than in-season (2208 kcal.day^−1^) and off-season (2161 kcal.day^−1^). The energetic intake of Brazilian players was examined on training day, match day and post-match day [40]. The intake was comparable on the training day (2386 kcal) and post-match day (2282 kcal), but it was significantly lower on match day (1940 kcal) [30].

**Table 2.** Studies that reported total energy intake in adult and youth female soccer players.

Study	Year	Group	N	Energy Intake (kcal.day^−1^)	
				mean	SD
Drobowolski et al.	2019	adult	21	1476	434
Dobrowolski et al.	2020	adult	31	1548	452
Pilis et al.	2019	adult	15	1714	335
Leão et al.	2022	adult	14	1764	495
Clark et al.^a^	2003	adult	13	1865	350
Łuszczki et al.	2021	adult	34	1872	255
Yli-Piipari	2019	adult	13	1894	428
Gonzalez-Neira et al.	2015	adult	17	1901	388
Martin et al.	2006	adult	16	1904	366
Morehen et al.	2022	adult	24	1923	357
Magee et al.	2020	adult	18	1931	371
Gonçalves et al.^b^	2015	adult	7	1940	489
Abood et al.	2004	adult	15	1969	414
Mullinix et al.	2003	adult	18	2015	19
Kang et al.^c^	2004	adult	14	2083	343
Behrens et al.	2020	adult	20	2112	505
Moss et al.	2021	adult	30	2124	444
Kang et al.^c^	2004	adult	11	2154	377
Reed et al.^d^	2013	adult	19	2161	143
Reed et al.^d^	2013	adult	19	2208	156
Fogellhom et al.	1995	adult	12	2251	401
Gravina et al.	2012	adult	28	2271	578
Gonçalves et al.^b^	2015	adult	7	2282	504
Clark et al.^a^	2003	adult	13	2290	310
Santos et al.	2016	adult	21	2306	405
Gonçalves et al.^c^	2015	adult	7	2386	639
Brinkmans et al.^e^	2023	adult	15	2344	
Dasa et al.	2023	adult	51	2274	450
Reed et al.	2013	adult	19	2794	233
Guzmán et al.	2011	adult	24	3050	140
McKinlay et al.	2022	youth	13	1622	139
Gibson et al.	2011	youth	33	2079	460
Cherian et al.	2018	youth	19	2243	320
Braun et al.	2018	youth	56	2403	195
Hosseinzadeh et al.	2017	youth	8	3122	476

SD (standard deviation); ^a^intake was detailed for pre-season and off-season; ^b^intake was detailed for match, post-match and training days; ^c^two groups were analyzed: supplementation and control; ^d^intake was detailed for pre-season, in-season and, off-season; ^e^authors did not present the SD.

**Figure 2.**
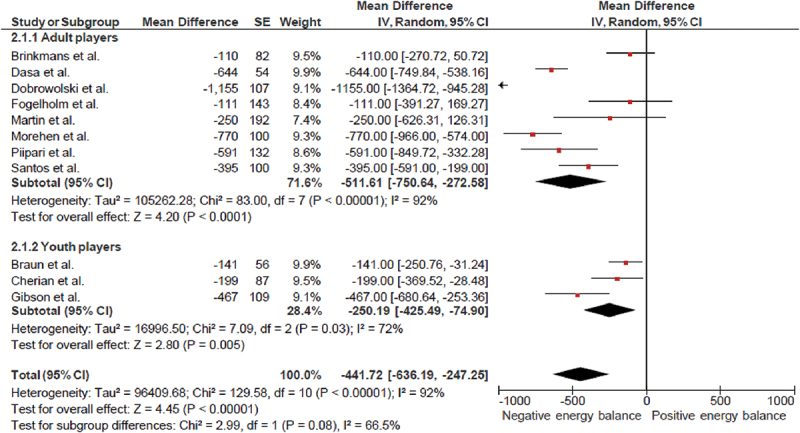
Forest plot of the differences between energy intake and energy expenditure.

Twelve studies reported the statistics (mean ± standard deviation) of daily energy intake and expenditure (Figure 2). In studies of adults [22,30,44,55,57,58,73,85,93] and youth players [32,52,53], raw mean differences between energy intake and total daily energy expenditure confirmed negative energy balance of – 441 kcal.day^−1^ (95% CI: – 636 kcal.day^−1^ to – 247 kcal.day^−1^). Some studies focused on the relationship between daily energy intake, exercise energy expenditure and body composition on energy availability [37,39,60,67].

Table 3 summarizes macronutrient intake in female youth and adult soccer players. Among adult players, relative CHO intake varied between 3.3 g.kg^−1^ to 7.0 g.kg^−1^, and protein ingestion ranged from 0.96 g.kg^−1^ to 2.0 g.kg^−1^. Among youth players, the values of protein ingestion were comparable in three different studies while mean values of CHO intake ranged from 5.0 g.kg^−1^ to 7.9 g.kg^−1^. Fourteen studies reported the relative fat intake, and mean values fluctuated from between 1.0 to 2.0 g.kg^−1^.

**Table 3.** Macronutrients intake in female soccer players.

Study	year	group	N	energy intake (g.kg^−1^)		
				CHO	protein	fat
Clark et al.^a^	2003	adult	14	5.2 ± 1.1	1.40 ± 0.30	
Clark et al.^b^	2003	adult	14	4.3 ± 1.2	0.96 ± 0.30	
Martin et al.	2006	adult	16	4.1 ± 1.0	1.2 ± 0.3	0.9 ± 0.2
Gibson et al.	2011	youth	33	5.0 ± 1.6	1.4 ± 0.3	1.2 ± 0.4
Reed et al.^c^	2014	adult	19	7.0 ± 1.0	2.0 ± 1.0	2.0 ± 1.0
Reed et al.^d^	2014	adult	19	5.0 ± 1.0	2.0 ± 1.0	1.0 ± 1.0
Reed et al.^e^	2014	adult	19	5.0 ± 1.0	1.0 ± 1.0	1.0 ± 1.0
Gonçalves et al.^f^	2015	adult	7	5.9 ± 2.0	1.9 ± 0.8	
Gonçalves et al.^g^	2015	adult	7	4.3 ± 1.0	1.5 ± 0.5	
Gonçalves et al.^h^	2015	adult	7	5.3 ± 1.3	1.9 ± 0.8	
Santos et al.	2016	adult	21	5.5 ± 0.9	2.0 ± 0.5	
Braun et al.	2018	youth	56	5.4 ± 1.1	1.4 ± 0.3	1.4 ± 0.4
Cherian et al.	2018	youth	19	7.9 (4.8–10.9)	1.5 (1.0–2.0)	0.78 ± 0.39
Drobowolski et al.	2019	adult	41	3.3 ± 1.2	1.2 ± 0.4	
Yli-Piipari	2019	adult	13	4.2 ± 2.3	1.6 ± 1.1	1.31 ± 0.89
Pilis et al.	2019	adult	15	3.6 ± 1.1	1.4 ± 0.4	
Magee et al.	2020	adult	18	3.7 ± 1.0	1.2 ± 0.3	1.1 ± 0.4
Moss et al.	2021	adult	31	3.3 ± 0.6	1.8 ± 0.4	1.3 ± 0.4
Morehen et al.	2022	adult	24	3.5 ± 0.9	1.9 ± 0.2	1.4 ± 0.3
Dasa et al.^c^	2023	adult	51	4.0 ± 1.3	1.6 ± 0.4	1.3 ± 0.4
Dasa et al.^d^	2023	adult	51	4.5 ± 1.9	1.7 ± 0.7	1.4 ± 0.7
Dasa et al.^i^	2023	adult	51	3.9 ± 1.6	1.5 ± 0.6	1.3 ± 0.7

^a^pre-season; ^b^off-season; ^c^training; ^d^match-day; ^e^post-match; ^f^pre-season; ^g^in-season; ^h^post-season; ^i^rest day.

Five studies focused on hydration status [35,43,48,63,65]. Overall, a large proportion of players tended to start soccer practice dehydrated. The percentage of players hypo hydrated ranged from 44─78% in 18 players from the under-19 Belgium team [48]. In the remaining studies, the prevalence of hypohydration before soccer sessions ranged from 45─54%.

## 4. Discussion

The present scoping review aimed to summarize existing scientific evidence investigating nutritional habits and practices in female soccer players. The main findings emerging in the current review include: (1) daily energy intake is widely variable across studies, (2) although different methods have been used to assess daily energy expenditure, a negative energy balance was consistent across studies, (3) the recommendations of CHO are rarely met by female soccer players while protein intake seems to be within the recommendations, (4) a significant percentage of female players ingested supplements, and (5) energy intake is the highest during preseason versus other phases of the year.

### 4.1 Energy balance and energy availability

The concept of energy balance reflects the difference between total daily energy intake and energy intake expenditure [11]. Energy deficit was found in the present review, which may have a negative impact on growth, health and performance [94]. Negative energy balance was highlighted in specific recommendations developed for youth athletes in such a way as to avoid long periods of energy restriction [95]. Many studies included the combination of youth and senior soccer players [23,45,55,71]. For instance, the age of Polish professional players ranged from 13 to 31 years old [55]. Given the differences in metabolic profiles between adults and youth populations [96], determining energy balance to formulate accurate nutritional plans remains a key challenge for nutritionists. Therefore, although using accurate techniques to estimate daily energy outputs of female soccer players may be logistically challenging, it is key that attempts are made to provide individualized targeted nutritional plans. Moreover, the studies included in the present scoping review were cross-sectional since they only described the total energy expenditure and energy intake of female players. The total energy expenditure represents the sum of resting energy expenditure, thermic effect of food, and exercise energy expenditure. Nutritionists often use equations to predict resting energy expenditure [97] based on a fixed physical activity coefficient multiplied by resting energy expenditure [98]. An additional challenge is the assessment of energy intake since the most common methods (i.e. weighted food records or food diaries) tend to underestimate the actual dietary intake [99]. Consequently, the mean values of total energy expenditure and energy intake reported are questionable, considering the error associated with these methodologies.

Although few studies in the present review compared changes in energy balance and body mass; one study reported a negative energy balance of – 825 kcal.day-1 across four days of assessment, but with negligible variations in body mass [75]. Therefore, this supports that energy balance should be measured over an extended period in conjunction with changes in body mass to be able to provide robust inferences that a negative energy balance has occurred. The principle of energy balance based on variation of body mass compartments (i.e. fat mass and fat-free) mass was recently described as useful to determine energy intake in the context of energy availability [10]. Energy availability is defined as the difference between energy intake and exercise energy expenditure once normalized for fat-free mass. This corresponds to the amount of dietary energy available for normal physiological functioning [100].

In the present review, nine studies assessed energy availability in soccer players [37,39,52,53,60,61,67,68,75]. Seven studies focused on examining the prevalence of low energy availability or associations between macronutrient intake and low energy availability [37,39,52,60,61,75]. In relation to the prevalence of low energy availability in female soccer players, the literature is inconclusive. In 19 American players aged 19-21 years, only five players (26%) were classified as low energy availability [39], whereas 88% of 24 professional international soccer players had < 30 kcal.kg_FFM_^.-1^ [75]. Different methodologies could explain the inconsistent findings to examine daily energy intake, exercise energy expenditure and body composition which define the concept of energy availability [101]. The evaluation of dietary intake was reported using multiple day food diaries [37,39,52,61,68]. Exercise energy expenditure was estimated based on exercise diaries [52,68], wearable devices [60,61] or based on total energy expenditure, resting energy expenditure and the thermic effect of food [71]. Fat-free mass and lean soft tissue were obtained by dual-energy X-ray absorptiometry [37,39,71,75], air-displacement plethysmography [61] or bioimpedance [52,60]. The pre-assessment conditions of air-displacement plethysmography and bioimpedance were not described in most of the studies. Unstandardized procedures (i.e. no control before the measurement) of these methodologies are a central factor in creating bias in body composition assessment [102]. The breakfast ingestion and daily activities were also reported as sources of error in measuring lean soft tissue in dual-energy X-ray absorptiometry scanning [103]. Indeed, variation of methodologies used to obtain fat-free mass are often ignored and may need to be corrected for accurate interpretations of energy availability [101]. Fluctuations in mean values of energy availability tend to occur across the season, which is affected by variations in exercise energy expenditure and daily energy intake during pre-season, in-season and off-season [37,101]. Meanwhile, acute periods of extreme energy deficits have no negative effects on performance if the players maintain an average energy availability within the adequate limits [101]. Nevertheless, players that were classified with low energy availability tended to exhibit a lower relative CHO intake [39,52,68]. Considering variations in training load and competitive schedules, maintaining an overall weekly energy availability within an acceptable interval and meeting the macronutrient recommendations is integral [101].

### 4.2 Soccer nutritional recommendations

Soccer-specific nutritional recommendations have not always differentiated between male and female athletes [94]. Based on training period and external load (duration, training distance, high-speed running), ranges of CHO have been provided for pre-season (4-8 g.kg^−1^), in-season with one match per week (3-8 g.kg^−1^), in-season with congested fixtures periods (6-8 g.kg^−1^) and off-season training (< 4 g.kg^−1^). Studies included in the present review showed mean values were nearer to the lower limit of CHO recommendations [55–57,61,68,75]. Female soccer players recognized the importance of fueling initial training but did not identify CHO as the primary energy source during exercise. Players tend to associate CHO intake with gains in fat mass and a negative appreciation of body shape [78]. One study found comparable values of mean relative CHO intake in pre-season, in-season and off-season [40]. Therefore, nutritionists should explain the importance of CHO intake to athletes and provide specific strategies to ensure CHO periodization considering training load and match demands [16]. The recommendations for CHO also focused on the hours before the match, during the match and post-match [94]. Two investigations included in the present review examined the importance of CHO ingestion before [70] and during the match [46]. Negligible differences in performance, perceptual changes, and physiological responses were found between high CHO and mixed macronutrient meals four hours before competition [70]. A study examined the effects of carbohydrate mouth rinsing (solution with 6% maltodextrin) in short-maximal outputs among 11 female soccer players [46]. Comparing CHO mouth rinsing and placebo trials, no differences in jump and velocity protocols were noted between conditions. Carbohydrate mouth rinsing is recommended for intermittent exercise given its benefits to sprint performance and purported lack of gastrointestinal symptoms [104,105]. However, given that CHO studies are often exclusive to male players, the form, quantity and timing of CHO in female soccer players require further attention.

To promote muscle skeletal adaptations and recovery, a protein intake of 1.6 to 2.2 g.kg^−1^ is recommended [94]. It has also been suggested that daily protein distribution (i.e. 20-30 g per meal) and quality are also crucial to optimize body composition [106]. In the present review, the mean values of daily protein ingestion were within the recommended values, but contrary to the literature on male soccer players, no studies have examined the protein intake throughout the day [107,108]. Among 14 adult Dutch elite male players, a skewed distribution of absolute protein intake across the day was noted, with significantly higher ingestions observed at breakfast, lunch and dinner compared to morning, afternoon and night snacks [107]. In youth female sports participants, protein intake recommendations tend to increase 2.3 g.day^−1^ especially during the maximal growth spurt [95]. In the three studies that included adolescent female players [32,52,53], the protein intake was below recommendations. This point is critical if the energy requirements are not met, potentially reducing protein availability for its primary functions of repair and regeneration [95].

Specific nutritional recommendations for female athletes considered the impact of the menstrual cycle [18]. It was suggested that exercise performance and muscle glycogen might be reduced during the early follicular phase (low levels of estrogen and progesterone) in comparison to the other phases of the menstrual cycle, with recommendations of 6-8 g.kg-1.day-1 during this phase [6,109]. However, studies on female soccer players have revealed inconsistent findings relating to the decrements in performance during the follicular phase and luteal phases [110-112]. Nine elite players covered 2883 m during the Yo-Yo intermittent recovery test during the mid-luteal phase (high levels of estrogen and progesterone), while players covered 3288 m during the follicular phase [111]. However, in a separate investigation, distance covered at very high intensities (16.69 km.h^−1^ to 19.94 km.h^−1^) tended to be higher in the luteal phase in contrast with the follicular phase [110]. Additionally, estrogen has been associated with anabolic (i.e. glucose uptake in the muscle and recovery) and progesterone on catabolic functions [113]. As such, nutritional guidelines for female soccer players should be carefully considered alongside the hormonal fluctuations associated with the menstrual cycle. Whilst specific nutritional recommendations for female athletes considering the influence of menstrual cycle require future research, practitioners may be advised to consider dietary modifications based on individual requirements and experiences.

Dehydration was frequently observed preceding soccer training or competition. Although slow rehydration is recommended by consuming 10 m^l.^kg^−1^ water 2–4 h prior to exercise [114], it is recommended that individualized interventions should be undertaken to examine hydration as well as personalized recommendations to attain hydration status before soccer training and competition [69]. However, the studies included did not use consistent methods, nor was care taken to standardized procedures across testing days. Therefore, further research is needed on hydration in female soccer players before hydration guidelines can be provided.

### 4.3 Limitations

A potential limitation of the studies included in the present scoping review is the heterogeneity of the methods used to quantify energy intake and energy expenditure. Food diaries often under-reported energy intake [115], while only one study examined energy expenditure using doubly-labeled water method [75]. The comparison of reference methods to evaluate total energy intake (i.e. food diaries, 24-hours recall) was contrasted with total energy expenditure measured by doubly labeled water in a meta-analysis that included 11 studies [99]. The overall energy balance in all studies was negative (mean value: 700 kcal.day^−1^; 19%) and differences between total energy intake and energy expenditure tend to increase with higher total energy expenditure. This systematic bias could be explained by general (e.g. body size, image, motivation, social expectations, the environment of assessment) and sport-specific factors (e.g. energy needs, frequency of meals, knowledge of portion sizes, commercial foods, supplements) that affect dietary intake [99,116]. Another possible limitation of the studies found in the present scoping review is the reporting of 24 hours of energy balance. This theory does not consider the endocrine responses associated with real-time changes in energy balance [117]. Consequently, continuous monitoring of energy balance measured in smaller intervals seems more appropriate since time spent in energy deficit was negatively associated with cortisol values in male and female endurance athletes [118,119].

## 5. Conclusion

The current study provides a broad overview of the nutritional practices of female soccer players. Female soccer players are predominately reported to be in negative energy balance, which may indicate that energy intake should be prioritized, with particular focus on CHO intake. Carbohydrate periodization has been largely examined in male soccer players, with few studies in females. The adjustment and planning of macronutrient intake among females needs to consider the periodization of the training load and match schedule. Nutritional intake, energy expenditure and body composition should be frequently assessed, given the risk of low energy availability in female players. Future studies should consider consistent methodological approaches to the measurement and reporting of nutritional intake. Additionally, more intervention-based studies would be a valuable addition to the literature as much of the current research is descriptive in nature. An emerging topic that requires further investigation is the impact of the menstrual cycle on the relationship between soccer training and competition and nutritional guidelines. Nutritional practices are a key avenue of research that must be explored to ensure that performance and health are optimized in female soccer.

## Data Availability

The data that support the findings of this study are available from the corresponding author, DVM, upon reasonable request.
